# Environmental and social factors impacting on epidemic and endemic tuberculosis: a modelling analysis

**DOI:** 10.1098/rsos.170726

**Published:** 2018-01-17

**Authors:** Chacha M. Issarow, Nicola Mulder, Robin Wood

**Affiliations:** 1 Computational Biology Division, Institute of Infectious Disease and Molecular Medicine, Faculty of Health Sciences, University of Cape Town, South Africa; 2 The Desmond Tutu HIV Centre, Institute of Infectious Disease and Molecular Medicine, Faculty of Health Sciences, University of Cape Town, South Africa

**Keywords:** infectiousness period, rebreathed air volume, effective contact number

## Abstract

Tuberculosis (TB) transmission results from the interaction between infective sources and susceptible individuals within enabling socio-environmental conditions. As TB is an airborne pathogen, the transmission probability is determined by the volume of air inhaled from an infected source and the concentration of *Mycobacterium tuberculosis* containing respirable particles (doses) per volume of air. In this study, we model the contributions of infectious dose production, prevalence of infectious cases and daily rebreathed air volume (RAV) for defining the boundary conditions necessary to sustain endemic TB transmission at the population level. Results suggest that in areas with high RAV (range 300–1000 l d^−1^), such as prisons, TB transmission is contributed by both super-spreaders (exhaling ≥10 infectious doses hr^−1^) and lower infectivity individuals (exhaling less than 10 infectious doses hr^−1^). In settings with a low quantity of RAV (less than 100 l d^−1^), TB transmission occurs only from super-spreaders. Point-source epidemics occur in low rebreathed environments when super-spreaders infect a number of susceptibles but subsequent transmission is limited by the mean infectivity of secondary cases. By contrast, endemic TB occurs in poor socio-environmental conditions where mean infectivity cases are able to maintain a sufficiently high effective contact number.

## 1. Introduction

Tuberculosis (TB) infection and disease vary markedly between different populations. In sub-Saharan Africa and many other developing countries, TB is endemic at a high level. The TB notification rate in South Africa is close to 1% per annum [1]. The TB notification rate in the HIV-negative population of Cape Town is comparable to rates recorded in the nineteenth century in industrialized settings [2]. Even within a highly endemic country, such as South Africa, pockets of extremely high TB prevalence continue in the gold mining [3] and prison populations [4,5]. In developed countries, endemic TB may be maintained within certain economically deprived sub-populations [6] and single source explosive outbreaks fail to develop into endemic TB [7].

Transmission of pulmonary TB results from the interaction between infective sources and susceptible individuals within enabling socio-environmental conditions. We have previously developed a model incorporating volume fraction of exhaled air and time of exposure to calculate daily rebreathed air volume (RAV) that reflects the social and environmental components of TB transmission, accounting for crowding and ventilation [8]. RAV has been measured in several TB transmission settings, which, in combination with the prevalence of infective cases and infectious particle (dose) production, allows for determination of TB transmission probabilities [9,8]. Social mixing and environmental conditions determine daily RAV [9]. Mean RAVs vary between 25 and 275 l d^−1^ for South African township adolescents [9] and are estimated to be greater than 1000 l d^−1^ for awaiting trial prisoners in an overcrowded South African prison [10].

Production rates of infectious doses vary widely. In a TB ward, infectious dose production was estimated at 1.25 doses hr^−1^ [11]. In a workplace outbreak from an untreated smear-positive pulmonary source, infectious dose production was estimated to be 12.7 doses hr^−1^ [12] and a production rate of 60 doses hr^−1^ has been reported from a highly infectious laryngeal TB case [13].

The period of infectiousness is impacted by the TB control programme that primarily identifies TB cases and initiates effective therapy, thereby decreasing prevalence of infective cases in the population. The mean period of infectivity can be defined by direct measurement [14] or estimated from TB prevalence divided by incidence rate [15]. The World Health Organization (WHO) global TB report 2015 estimated South African prevalence and incidence rates at 696/100 000 and 834/100 000, respectively, which is consistent with a mean period of infectiousness of 10 months [1]. Additionally, directly measured period of infectiousness of TB patients before treatment vary between 0.73 years (8.76 months) and 1.02 years (12.24 months) for HIV-negative, smear negative and smear positive in South African population [14].

For TB to remain endemic in any population, the case reproductive number must be equal to or exceed 1 [16]. The reproductive number is markedly influenced by the lifetime risk of progression from infection to active smear-positive disease that has been estimated to be 5–10% [17,18]. The number of secondary cases infected by a TB case (effective contact number) would therefore be required to equal or exceed 20 during the mean period of infectiousness prior to initiation of effective therapy.

The factors contributing to TB transmission probability, including RAV and source infectivity, are continuous variables, and it is therefore not possible to define absolute threshold values beneath which risk of transmission cannot occur. However, these variables in combination define the boundary conditions necessary to create sufficient secondary cases to sustain endemic TB. In this study, we model the contributions of infectious dose production, prevalence of infectious cases and RAV for defining the boundary conditions necessary to sustain endemic TB transmission. The model outputs are used to identify socio-environmental and TB control programme values required to interrupt endemic TB.

## 2. Methods

### 2.1. Modelling

To explore the components contributing to TB transmission and quantify the boundary conditions necessary to sustain endemic TB using a mathematical modelling approach, we use real-world values of effective contact number of 20 and 100, RAV ranging from 0 to 1000 l d^−1^, surviving airborne infectious doses between 1 and 30 doses hr^−1^, alveoli deposition fraction of 0.1 and period of infectiousness ranging from 0 to 14 months as described in table 1. In special cases, we use fixed values of infectiousness period of 10 months and RAV of 100 l d^−1^ to observe the proportions of susceptible individuals infected for source infective dose production between 1 and 30 doses hr^−1^. As mentioned earlier, the production rates of infectious doses vary widely. We therefore define super-spreaders as efficient transmitters or infectious individuals exhaling ≥10 infectious doses hr^−1^ and lower infectivity for those exhaling less than 10 infectious doses hr^−1^.

**Table 1.** Description of parameters and values used in this study for numerical simulations.

parameter	description	values	sources
△	period of infectiousness	0–14 months (10 months for special cases)	[1,14]
*M*	TB incidence in South Africa	834 per 100 000 population	[1,19]
*P*_V_	TB prevalence in South Africa	696 per 100 000 population	[1]
ECN	effective contact number per TB cases	20 and 100	[20,21]
*β*−*μ*	surviving airborne infectious doses	1–30 doses hr^−1^	modelled
*θ*	alveoli deposition fraction	0.1	[22]
RAV	rebreathed air volume	0–1000 l d^−1^ (100 l d^−1^ for special cases)	[9,10]

The design and development of the model is based on the socio-environmental components influencing endemic TB transmission in high TB settings, such as Cape Town where TB notification rate among HIV-negative population is remarkably high [2]. We take into account that when susceptible individuals become exposed and airborne infectious doses viable with the potential for TB infection reach the alveoli, they may be (re)infected or not, depending on the virulence of the infecting pathogen strain and the immunological status of the hosts [8]. Thus, we explore socio-environmental conditions contributing to TB transmission and quantify the boundary conditions required to sustain endemic TB by considering and extending the mathematical model developed in Issarow *et al.* [8] that predicts the risk of airborne infectious diseases under steady-state and non-steady-state conditions as follows:

2.1 $$\frac{C}{S}=1-e^{-I(\beta -\mu )\theta pt/Q},$$

where *C* denotes the number of new TB cases, *S* is the number of susceptible individuals, *Q* is the ventilation flow rate (l hr^−1^), *p* is the breathing rate (l hr^−1^), *I* is the number of infectious individuals in the space, *θ* is the deposition fraction of infectious doses in the alveoli, *t* is the duration of exposure (hr) and (*β*−*μ*) is the surviving airborne infectious doses per unit time (doses hr^−1^) that reach the alveoli to establish infection depending on the virulence of the infecting pathogen strain and host immune systems [8]. *β* is the total number of airborne infectious dose production per unit time (doses hr^−1^) and *μ* is the mortality rate of airborne infectious doses (doses hr^−1^) before reaching the target infection site of the host.

We take into consideration that the number and concentration of viable airborne infectious doses with the potential for TB infection depends on air volume in the given space. Hence, considering the production of airborne infectious doses per air volume (doses l^−1^), while other parameters remain constant, equation (2.1) becomes

2.2 $$\frac{C}{S}=1-e^{-I(\beta -\mu )\theta p^{2}t/Q}.$$

The fractions of breathed and rebreathed air are correlated as follows:

2.3 $$\frac{p}{Q}=\frac{f}{n},$$

where *f* is the fraction of rebreathed air and *n* is the total number of individuals in the space.

Substituting *f*/*n*=*p*/*Q* from equation (2.3) into equation (2.2), gives

2.4 $$\frac{C}{S}=1-e^{-I(\beta -\mu )\theta fpt/n}.$$

Prevalence is one of the most significant factors used to measure TB risk [23,24]. As mentioned earlier, the mean period of infectivity can be measured directly [14] or estimated from the ratio of TB prevalence and incidence rate [15]. The WHO global TB reports show that the period of infectiousness in South Africa is 10 months, which was computed as the ratio of TB prevalence and incidence rate [24]. In equation (2.4), *I*/*n* (TB cases/total number of population) denotes TB prevalence (*P*_V_), which can also be computed as the product of incidence rate (*M*) and period of infectiousness (△) [23], such that

2.5 $$P_{V}=M\times △.$$

Therefore, in terms of prevalence as the product of incidence and period of infectiousness, equation (2.4) becomes

2.6 $$\frac{C}{S}=1-e^{-M△(\beta -\mu )\theta fpt}.$$

RAV is the product of breathing rate (*p*), rebreathed air fraction (*f*) and duration of exposure (*t*) [9], such that

2.7 RAV=fpt.

Substituting RAV from equation (2.7) into equation (2.6) gives an equation that incorporates RAV to predict the risk of airborne infectious diseases as:

2.8 $$\frac{C}{S}=1-e^{-M△(\beta -\mu )\theta (\mathrm{RAV})}.$$

Simulating equation (2.8) numerically, we obtain the proportion of susceptible individuals infected (*C*/*S*) as a function of RAV and period of infectiousness for variable surviving airborne infectious doses as discussed in the results.

The number of new TB cases can be computed as the product of effective contact number (ECN), infectious individuals (*I*) and susceptible individuals (*S*) [25], such that

2.9 C=(ECN)IS.

Dividing by *S* both sides in equation (2.9) leads to

2.10 $$\frac{C}{S}=\left(\mathrm{ECN}\right)\text{I},$$

which is equivalent to equation (2.8). Thus, substituting (ECN)*I*=*C*/*S* from equation (2.10) into equation (2.8) gives

2.11 $$\left(\mathrm{ECN}\right)\text{I}=1-e^{-M△(\beta -\mu )\theta (\mathrm{RAV})}.$$

Introducing natural logarithm in equation (2.11) and simplifying further by making RAV the subject, we obtain

2.12 $$\mathrm{RAV}=\frac{\ln{\left(1-\left(\mathrm{ECN}\right)\text{I}\right)}^{-1}}{M△(\beta -\mu )\theta },\;I\geq 1.$$

Recently published studies show that the ECN of 20 and 100 sustain endemic TB in both fully susceptible and latently infected populations [20,21]. The ECN of 20 sustains endemic TB for the susceptible population [20] and that of 100 for the population with latent TB infection [21]. It is estimated that the risk of developing active disease for the latently infected is 79% lower than uninfected individuals [21], This would represent an approximately ECN of 100 for previously infected individuals. Thus, in this study we use the ECN of 20 and 100 to determine socio-environmental conditions required to sustain endemic TB transmission in a variety of settings. Additionally, since HIV increases disease progression and decreases the ECN on population tuberculosis [26], we modelled the impact on an HIV-infected population using the ECN of 10. Using modelled infective dose production and real-world values described in table 1 in equation (2.8), we explored socio-environmental factors impacting on TB transmission probability. Furthermore, we quantified the boundary conditions necessary to sustain endemic TB by simulating equation (2.12) numerically as discussed in the results. Parameters and values used in this study for numerical simulation analysis are described in table 1.

## 3. Model limitations

Our transmission model is a development of the Wells and Riley equation that defined the physical factors that contribute to TB transmission risk of susceptible individuals exposed for a fixed time period to an infectious source within a steady-state environment. Our parameter of daily-rebreathed indoor air volume, which has been directly measured in a South African population, allowed capture of potential environmental transmission risk for individuals traversing multiple indoor locations without the necessity to define the ventilation within each location. Our model is based on a finding that ‘social characteristics operate only as they contribute to the environmental factor in transmission of TB infection’ [27]. We populated the model with a wide range of infectivity and period of infectiousness, as there are limited data describing these parameters at a population level. We focused on a population with a high level of endemic TB transmission; however, populations are not homogeneous and transmission risks vary between subgroups within each population. Furthermore, as each of the parameters in the model are continuous variables which interact with each other, we were not able to define programmatic or environmental thresholds above which reduced average transmission will not sustain endemic. We used our model to demonstrate interaction for values of those variables in combination that no longer exceed the ECN required to sustain endemic TB transmission. Our model incorporated change in risk of progression for latently infected individuals; however, the role of multiplicity of infections and age at infection was not addressed.

## 4. Results

Using equation (2.8), we substituted modelled infectious dose production and real-world values for the above variables in order to explore how socio-environmental conditions (RAV) and TB control programme (period of infectiousness) affect the proportions of infections among a population of susceptible individuals.

**Figure 1.**
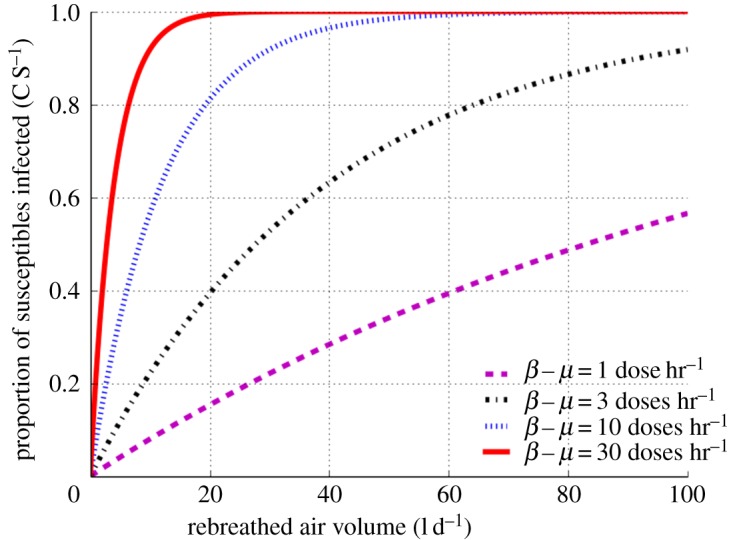
Proportion of susceptible individuals infected as a function of daily rebreathed air volume. Data shown for source infective dose production between 1 and 30 doses hr^−1^ using a fixed infectiousness period of 10 months.

Firstly, we explored the impact of RAV on TB transmission probability while maintaining a constant period of infectiousness of 10 months for individuals with infective dose production between 1 and 30 doses hr^−1^. Figure 1 shows that transmission probabilities reach 100% at RAV 20 and 60 l d^−1^ for super-spreaders exhaling ≥10 infectious doses hr^−1^. By contrast, individuals with lower infectivity (exhaling less than 10 doses hr^−1^) do not reach transmission saturation at RAV less than 100 l d^−1^.

**Figure 2.**
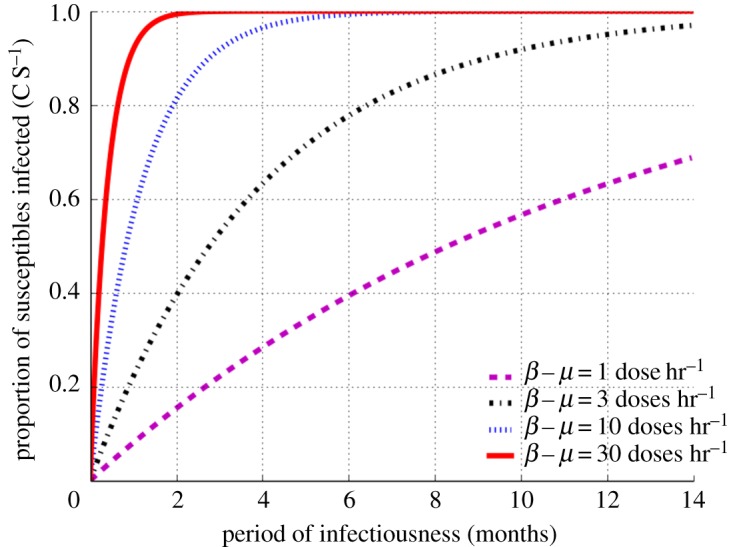
Proportion of susceptible individuals infected as a function of infectiousness period. Data shown for source infective dose production between 1 and 30 doses hr^−1^ using a fixed rebreathed air volume of 100 l d^−1^.

Secondly, we went on to explore the impact of period of infectiousness on transmission probability while maintaining RAV at 100 l d^−1^. Figure 2 shows that transmission probabilities reach 100% at periods of infectiousness of two and six months for super-spreaders exhaling ≥10 infectious doses hr^−1^. By contrast, individuals with lower infectivity do not reach transmission saturation for periods of infectiousness up to 14 months. Although figures 1 and 2 have different scales and parameters, the overall patterns for individuals at each different level of infectivity are very similar, demonstrating that both RAV (socio-environmental) and period of infectiousness (TB control programme) are important determinants of TB transmission.

**Figure 3.**
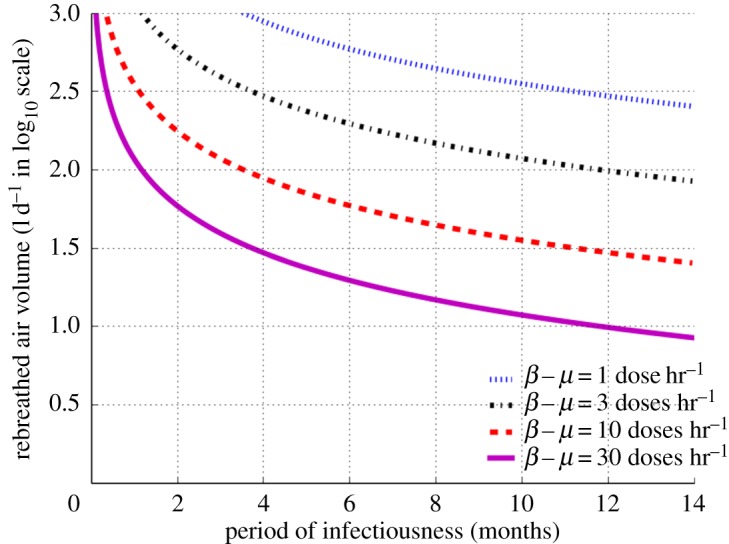
Quantity of daily rebreathed air volume in $\log_{10}$ scale as a function of infectiousness period for effective contact number of 20 and source infective dose production between 1 and 30 doses hr^−1^.

Next we used equation (2.12) for exploration of the interactions between RAV and period of infectiousness using defined boundary conditions of effective contact number (ECN) of 20 for a fully susceptible population [28,29]. To enable clear separation of curves across the very wide range of RAV we expressed the vertical axis (ordinate) in $\log_{10}$ scale (figure 3). We noted that in order to decrease ECN to less than 20, both RAV and period of infectiousness must be reduced. In order to reduce ECN to less than 20 for super-spreaders (exhaling ≥10 infectious doses hr^−1^), RAV needs to be as low as 30−100 l d^−1^ and period of infectiousness reduced to less than six months. By contrast, to reduce ECN to less than 20 for lower infective cases (exhaling less than 10 infectious doses hr^−1^), RAV is only required to be less than 300 l d^−1^ and the period of infectiousness reduced to less than 14 months.

**Figure 4.**
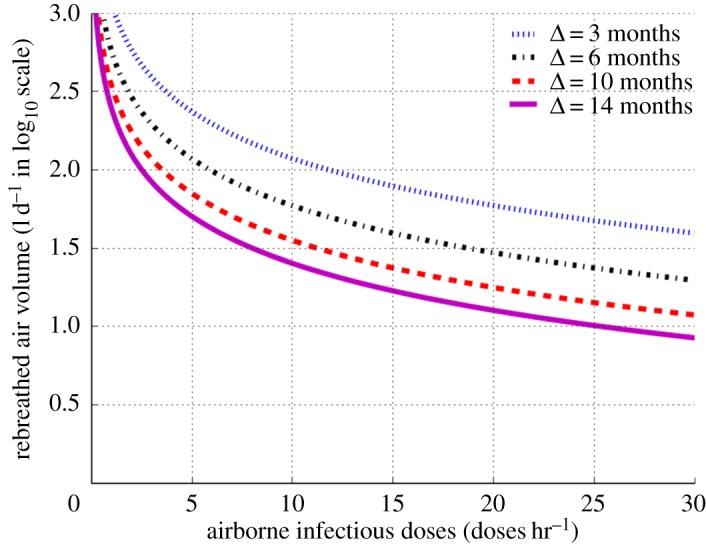
Quantity of daily rebreathed air volume in $\log_{10}$ scale as a function of airborne infectious doses for effective contact number of 20 and period of infectiousness ranging between 3 and 14 months.

We then went on to explore the relationship between infectious dose production and RAV required to maintain the ECN of 20 in figure 4. Similarly, to enable clear separation of curves across the very wide range of RAV we expressed the ordinate in $\log_{10}$ scale. At infectious dose production rates greater than 10 doses hr^−1^ (super-spreaders), the slopes of the graphs are nearly horizontal for each of the periods of infectiousness (3–14 months) such that transmission would reach the ECN of 20 when RAV is between 10 and 100 l d^−1^. Super-spreaders can therefore transmit over the range of RAVs that would exist in most industrialized settings. By contrast, the slopes increase as infectivity diminishes such that for infectious dose production less than 3 doses hr^−1^, transmission occurs only between 100 and 1000 l d^−1^. Under poor socio-environmental conditions (high RAV), lower infectivity individuals contribute to endemic TB transmission.

The findings in this study indicate that in areas with high RAV (range of 300–1000 l d^−1^), such as prisons, TB transmission is contributed by both super-spreaders and lower infectivity individuals. In settings with low RAV (less than 100 l d^−1^), sustainable endemic TB transmission occurs from super-spreaders, while lower infectivity individuals fail to sustain endemic transmission though they may transmit after a long period of infectiousness (more than six months). A high value of RAV was observed in the regions with a small number of surviving airborne infectious doses, between 1 and 10 doses hr^−1^, suggesting that endemic TB occurs in poor socio-environmental conditions where mean infectivity cases are able to maintain a sufficiently high effective contact number.

## 5. Discussion

Our analysis is predicated on the concept that the probability of acquiring TB infection is determined by the risk of exposure to an infective dose. Studies in the 1950s defined the relationship between ventilation and the concentration of infective organisms in air within a single steady-state location [11]. We previously extended TB transmission probability analysis to non-steady-state conditions prevailing in TB endemic settings [8]. The volume of air exchanged between individuals is related to the prevailing socio-environmental conditions and the prevalence of TB infectious individuals is determined by the TB control programmes’ ability to identify and effectively treat infective TB cases in the population. In this analysis, we explored the boundary conditions determined by volume of exchanged air, mean infective period and infectivity required to sustain endemic TB transmission in both fully susceptible and latently infected populations. Our modelling analysis initially showed that exchanged air volume and period of infectiousness of TB cases are both equally important drivers of TB infection risk. Recently published studies using carbon dioxide as a naturally occurring tracer gas have measured the volume of exchanged air among residents of South African townships where TB is endemic to be in the range of 100–300 l d^−1^ [9] and has been estimated to reach greater that 1000 l d^−1^ in a crowded prison population [10]. When air volumes exchanged between individuals are high, most TB cases, even those with low infectivity are able to transmit disease to sufficient numbers of susceptible and latently infected individuals, thus maintaining endemic TB. Indoor carbon dioxide values are directly related to per-person ventilation and 1000 ppm (600 ppm above environmental) represents a mean per-person ventilation of 666 l min^−1^ [8]. Under extremely poor socio-environmental conditions, such as exist in South African prisons, the TB control programme is very unlikely to be able to sufficiently decrease the prevalence of TB infectious individuals to reduce endemic TB transmission without concurrent improvements in prevailing overcrowding and environmental conditions [10]. By contrast, when exchanged volumes of air are less than 100 l d^−1^, a TB control programme can easily limit transmission to only the most infectious individuals exhaling greater than 10 infectious doses hr^−1^. Super-spreader transmission in reasonable socio-environmental conditions would therefore be projected to result in an explosive point-source transmission outbreak that could not subsequently be sustained by secondary transmission.

This analysis is focused on TB transmission rather than progression to TB disease, which is impacted by many other factors such as vitamin D status and co-morbidities such as diabetes and neoplastic disease. However, control of acquisition of infectious disease by environmental control measures or vaccination has been fundamental to disease control. Our study quantifies the contribution of socio-environmental factors in addition to the effective identification and treatment of TB cases and demonstrates that the effectiveness of a TB control programme is markedly modified by prevailing socio-environmental conditions. Importantly, the demonstration of the synergistic interaction between RAV and TB prevalence for the reduction of the effective contact number required to maintain endemic TB does offers an additional strategy for TB control. The current Stop TB strategy focuses on integrated patient-centred care and prevention, and health system strengthening [30] to decrease TB prevalence, but does not directly address the socio-environmental conditions that maintain TB transmission in highly endemic populations. The volume of rebreathed air in any indoor environment is directly proportional to prevailing carbon dioxide levels [9,31], a measure of per-person ventilation that can be readily monitored. In order to reach the sustainable development goal of 90% decrease in TB deaths and 95% decrease in TB incidence by 2035 [30] our analysis indicates that maintaining a carbon dioxide level at less than 1000 ppm in the major transmission hotspots such as public transport [32], schools [33] and prisons [10] would reduce RAV to 12.5 l hr^−1^, which would be sufficient to interrupt endemic TB transmission. Public health interventions to reduce transmission of TB should act synergistically with existing TB control programmes, which have failed to control TB in current high TB settings, such as South Africa [34].

**Figure 5.**
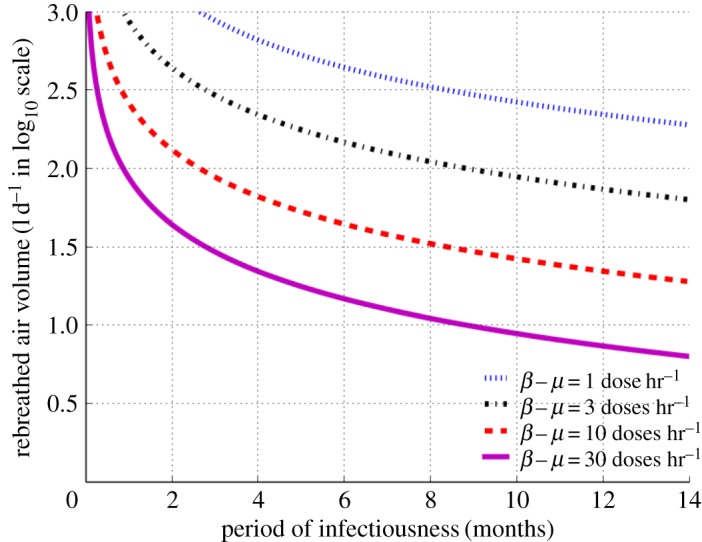
Quantity of daily rebreathed air volume in $\log_{10}$ scale as a function of infectiousness period for effective contact number of 10 and source infective dose production between 1 and 30 doses hr^−1^.

**Figure 6.**
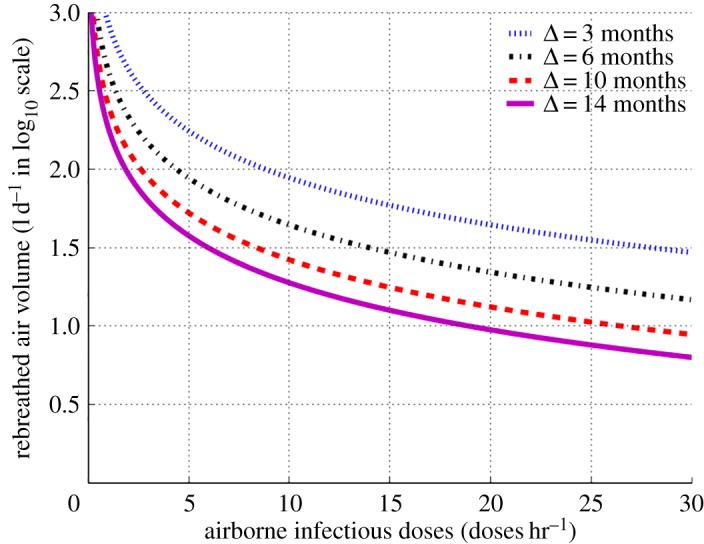
Quantity of daily rebreathed air volume in $\log_{10}$ scale as a function of airborne infectious doses for effective contact number of 10 and period of infectiousness ranging between 3 and 14 months.

HIV increases the progression from infection to disease, especially when the CD4 counts are low, and is partially reversed with antiretroviral therapy [26]. The impact of HIV on population tuberculosis will decrease the ECN within the HIV-infected. The impact on the HIV-infected population is modelled in figures 5 and 6 (appendix A) using the ECN of 10. In order to reduce the ECN to less than 10, the RAV needs to be as low as 20–80 l d^−1^ for super-spreaders and period of infectiousness reduced to less than four months. For all levels of infectiousness and RAV, control of endemic TB requires the period of infectiousness to be reduced, highlighting the need for active case finding in the HIV-infected population. However, the impact on transmission within the total population will be limited by the prevalence of HIV, which currently in South Africa is 18% of the adult population, and access to antiretroviral therapy [35].

## Figures and Tables

**Figure 7.**
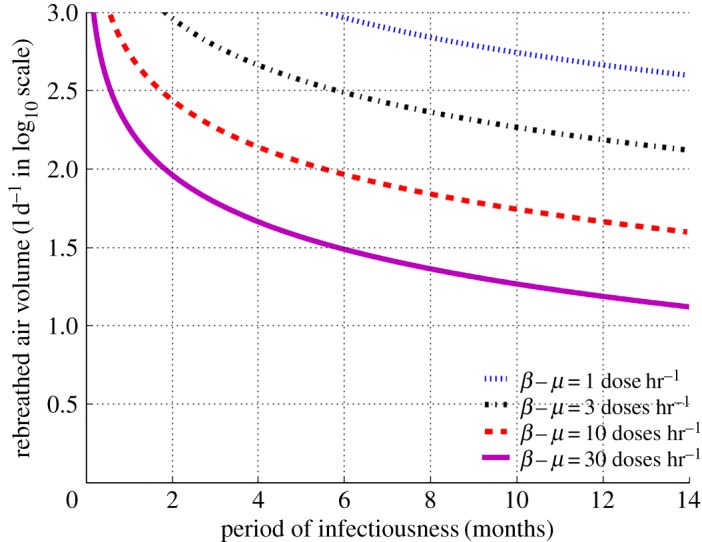
Quantity of daily rebreathed air volume in $\log_{10}$ scale as a function of infectiousness period for effective contact number of 100 and source infective dose production between 1 and 30 doses hr^−1^.

**Figure 8.**
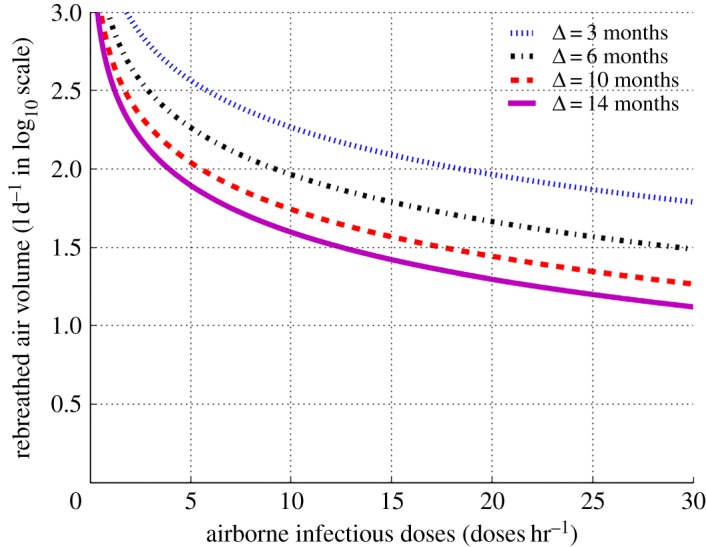
Quantity of daily rebreathed air volume in $\log_{10}$ scale as a function of airborne infectious doses for effective contact number of 100 and period of infectiousness ranging between 3 and 14 months.

## Data accessibility

All the data sources used in the study analysis are referenced in table 1.

## Appendix A

Further analysis explored the relationship between causative variables necessary to sustain endemic TB in a previously infected (latent) population. It has been suggested that primary TB infection may protect against subsequent infection progressing to active disease [21]. The estimated protection of 79% would translate into a required ECN of close to 100 to maintain endemic TB in a latently infected population [21]. Figure 7 shows the relationship between RAV and period of infectiousness for boundary conditions of ECN of 100. Figure 8 shows the relationship between RAV and infectious dose production for boundary conditions of ECN of 100. Figures 7 and 8 do not differ markedly from figures 3 and 4, indicating that the protection afforded by latent infection impacts minimally on the transmission dynamics required to sustain endemic TB.

As HIV accelerates disease progression and decreases the ECN, we demonstrated the impact on the HIV-infected population using the ECN of 10 in figures 5 and 6. The ECN of 20 and 100 for HIV-negative seem to have topological similarities with that of HIV-positive at the ECN of 10, implying that reduction of the period of infectiousness and RAV is crucial for HIV and TB control programmes.
